# Structural basis for hypermodification of the wobble uridine in tRNA by bifunctional enzyme MnmC

**DOI:** 10.1186/1472-6807-13-5

**Published:** 2013-04-24

**Authors:** Jungwook Kim, Steven C Almo

**Affiliations:** 1 Albert Einstein College of Medicine, 1300 Morris Park Avenue, Bronx, New York 10461, USA

**Keywords:** tRNA modification, X-ray crystallography, SAM-dependent methyl transferase, Oxidoreductase

## Abstract

**Background:**

Methylaminomethyl modification of uridine or 2-thiouridine (mnm5U34 or mnm5s2U34) at the wobble position of tRNAs specific for glutamate, lysine and arginine are observed in *Escherichia coli* and allow for specific recognition of codons ending in A or G. In the biosynthetic pathway responsible for this post-transcriptional modification, the bifunctional enzyme MnmC catalyzes the conversion of its hypermodified substrate carboxymethylaminomethyl uridine (cmnm5U34) to mnm5U34. MnmC catalyzes the flavin adenine dinucleotide (FAD)-dependent oxidative cleavage of carboxymethyl group from cmnm5U34 via an imine intermediate to generate aminomethyl uridine (nm5U34), which is subsequently methylated by S-adenosyl-L-methionine (SAM) to yield methylaminomethyl uridine (mnm5U34).

**Results:**

The X-ray crystal structures of SAM/FAD-bound bifunctional MnmC from *Escherichia coli* and *Yersinia pestis*, and FAD-bound bifunctional MnmC from *Yersinia pestis* were determined and the catalytic functions verified in an *in vitro* assay.

**Conclusion:**

The crystal structures of MnmC from two Gram negative bacteria reveal the overall architecture of the enzyme and the relative disposition of the two independent catalytic domains: a Rossmann-fold domain containing the SAM binding site and an FAD containing domain structurally homologous to glycine oxidase from *Bacillus subtilis*. The structures of MnmC also reveal the detailed atomic interactions at the interdomain interface and provide spatial restraints relevant to the overall catalytic mechanism.

## Background

tRNAs are the most frequently modified cellular RNAs in all three phylogenetic domains of life. To date, almost 100 modified nucleosides have been reported in the tRNA sequence database (http://rna-mdb.cas.albany.edu/RNAmods/). Position 34 in tRNAs, the 5′ nucleoside of the anticodon triplet, also known as the *wobble* position, exhibits the greatest propensity for post-transcriptional modification, with nearly 50% of all *E. coli* tRNAs bearing modifications at this site [1]. Modifications at the wobble position affect the codon recognition properties of the tRNA and are essential for the accurate and complete reading of the genetic code [2]. The wobble hypothesis was proposed by Crick to account for the observation that most organisms code for considerably fewer tRNAs than the number of sense codons, precluding a simple one-to-one correspondence between codon and tRNA. Specifically, he postulated that the first nucleoside of the anticodon is less constrained than the last two, which results in the required degeneracy by allowing non-canonical recognition (*i.e.*, non-Watson-Crick base pairing) of the last nucleoside of a codon presented on the ribosome [3].

Accumulating tRNA sequence data and increased knowledge of the chemical structures of modified nucleosides led to a modified wobble hypothesis, which encompasses the effects of post-transcriptionally modified uridines frequently found at the anticodon wobble position in bacteria and eukaryotic [4,5]. For example, oxyacetyl uridine (cmo5U34) at the wobble position allows tRNA^Val^ and tRNA^Pro^ to recognize all four bases in the 3′ position of the codon, while modification of U34 to 5-methylaminomethyl-2-thiouridine (mnm5s2U34) enables tRNA^Glu^ and tRNA^Lys^ to read codons ending in A or G but not C or U [6]. In addition, 5-methylaminomethyl uridine (mnm5U34), which lacks thiolation at C2 of the pyrimidine, permits tRNA^Arg^ to decode AGA and AGG, but not AGC and AGU.

**Figure 1:**
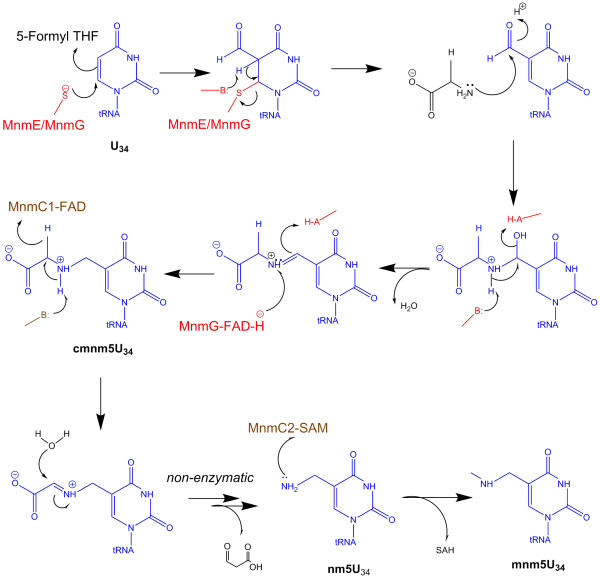
**Proposed reaction mechanism for the biosynthetic pathway of mnm5U34. **In the first step, the MnmE/MnmG protein complex catalyzes the fusion of formyl group from 5-formyl tetrahydrofolate (THF) and the wobble uridine (U34) of tRNA. The amino group of glycine attacks the carbonyl group of formyl-tRNA to yield a Schiff base, which is reduced by MnmG-bound FADH. Carboxymethylaminomethyl uridine (cmnm5U) is oxidized between Cα-N bond by MnmC1-bound FAD. The resulting imine intermediate is non-enzymatically hydrolyzed to yield aminomethyl uridine (nm5U), which is then methylated by SAM in the MnmC2 active site.

In *E. coli*, the enzymes involved in biosynthesis of the thiol-bearing mnm5s2U34 have been identified; MnmA (formerly AsuE or TrmU), together with the cysteine desulfurase IscS, catalyze the thiolation of position 2 of the wobble uridine, leading to 2-thiouridine (s2U) on tRNA^Glu^ and tRNA^Lys^[7]. MnmE and MnmG form an α_2_β_2_ heterotetramer [8,9] that catalyzes transformation of the wobble uridine and s2U34 to 5-carboxymethyl aminomethyl uridine (cmnm5U34) and 5-carboxymethylaminomethyl-2-thiouridine (cmnm5s2U34), respectively. These modifications at 2- and 5- positions have been shown to occur independently to each other [10]. MnmC (formally known as YfcK or TrmC) is a bifunctional enzyme responsible for the final two steps of biosynthetic pathway of mnm5s2U in tRNA^Glu^ and tRNA^Lys^, and mnm5U in tRNA^Arg^[11,12]. As illustrated in Figure 1, the C-terminal domain (MnmC1) catalyzes the flavin adenine dinucleotide (FAD)-dependent oxidation of the C_α_-N bond in cmnm5U34. The resulting imine intermediate is (presumably) non-enzymatically hydrolyzed to 5-aminomethyl uridine (nm5U34), followed by S-adenosyl L-methionine (SAM)-dependent methylation to yield mnm5U in the N-terminal domain active site (MnmC2). Bifunctional MnmC is found predominately in γ-proteobacteria, while non-fused orthologs of the MnmC1 and MnmC2 domains are present in various other bacteria [12]. Although MnmC is not essential for *E. coli*, the ΔMnmC strain exhibits a slower growth rate compared to the wild-type, suggesting that cmnm5U34 is not as efficient as mnm5U34 in supporting translation [13].

To date, crystal structures have been reported for the FAD-bound bifunctional MnmC (3AWI), the isolated MnmC2 domain (i.e., a truncated MnmC from *E. coli;* 2QY6) and the stand-alone MnmC2 from Aquifex aeolicus (3VYW). Here we present the FAD/SAM-bound crystal structures of full-length MnmC from *E. coli* (ecMnmC) and *Y. pestis* (ypMnmC), and an FAD-bound crystal structure of ypMnmC. These are the first experimentally determined structures of bifunctional MnmC with both FAD and SAM bound, and reveal the architectures of both active sites, as well as the detailed interactions between the MnmC1 and the MnmC2 domains. These structures also suggest features of MnmC catalysis that will aid further kinetic and mechanistic investigations.

## Results

### MnmC is monomer in solution

The oligomeric state of MnmC was examined by size exclusion chromatography. Under the conditions employed, ypMnmC and ecMnmC eluted from a Superdex75 column at 52.2 mL and 52.3 mL, respectively (Additional file 1: Figure S1). Bovine albumin (molecular weight of 66 kDa) eluted at 53.5 mL, close to the size of monomeric MnmC, ~75 kD; γ-globulin (molecular weight of 158 kDa), which approximates the molecular weight of a putative MnmC dimer, eluted considerably faster at 47.8 mL. These data are consistent with a model in which MnmC exists as a monomer in solution.

### Functional assay of MnmC

To confirm that our recombinant MnmC utilizes cmnmU34-tRNA as a substrate, post-transcriptionally modified tRNA^Arg^ substrate was generated *in situ* by incubating *in vitro* transcribed pre-tRNA^Arg^ with MnmE and MnmG, as well as the necessary substrates including glycine, NADH, 5-formyl-tetrahydrofolate (THF), and GTP, in addition to ^14^C-labeled SAM and MnmC (Figure 1). After quenching the reaction with 0.5% TCA, the precipitated RNAs were bound on an ion exchange cellulose filter, which was washed extensively with 0.5% TCA and examined for retention of radioactivity. The dual activity of *E. coli* and *Y. pestis* MnmC was confirmed by the incorporation of ^14^C-label on the modified tRNA as shown in Additional file 1: Figure S2.

### Overall structure of MnmC

**Figure 2:**
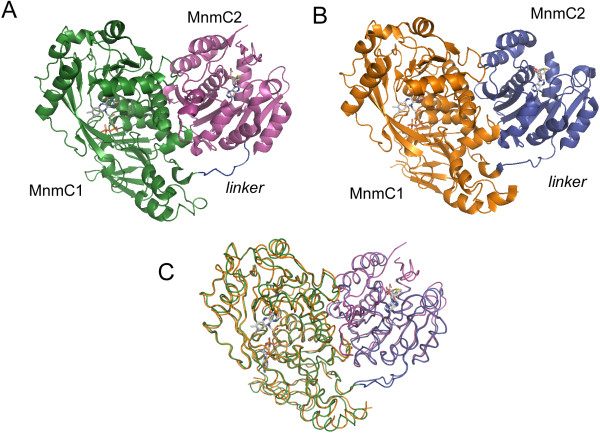
**Structural overview of ecMnmC and ypMnmC.**  Ribbon representation of the overall structure of **A**) ecMnmC, where the MnmC1 and MnmC2 domains are colored green and purple, respectively and **B**) ypMnmC, where MnmC1 and MnmC domains are orange and blue. The ‘linker’ domain is indicated as defined in the text. FAD and SAM are displayed in sticks, where carbon atoms are in grey, oxygen atoms in red, nitrogen atoms in blue, and sulfur atoms in yellow. **C**) Overlay of ecMnmC and ypMnmC structures, where the same color scheme is used as in **A**) and **B**).

Each MnmC crystal structure contains a monomer in the asymmetric unit (crystallographic statistics are summarized in Table 1). Consistent with the observed monomeric state of MnmC in solution, no significant oligomeric interfaces could be detected between symmetry related molecules in the two crystal forms examined. In the FAD- and FAD/SAM-bound ypMnmC structures, of the 689 residues, the first 28 N-terminal residues, 183–184, 451, and 602–609 are not represented by electron density. In the ecMnmC structure, out of 668 residues, only residues 15–17 and 664–668 could not be modeled. All three structures of bifunctional MnmC exhibit a similar organization with the MnmC1 and MnmC2 domains being connected by a stretch of ~10 amino acids (Glu-245-Pro-255 in ecMnmC and Pro245-Pro254 in ypMnmC) (Figure 2).

**Table 1:** Crystallographic data

**Enzyme**	**FAD/SAM-ecMnmC**	**FAD- ypMnmC**	**FAD/SAM-ypMnmC**
**Data Collection**			
Space Group	P41212	P21	P21
Cell Dimension			
a, b, c (Å)	100.06, 100.06, 159.19	65.15, 59.57, 99.63	66.09, 59.82, 100.80
α, β, γ (°)	90, 90, 90	90.00, 99.57, 90.00	90.00, 100.18, 90.00
Resolution (Å)	50.0–2.55 (2.64-2.55)	50.0–2.30 (2.37-2.30)	50.0–2.70 (2.75-2.70)
I/σ	9.9 (2.5)	14.8 (2.6)	9.3 (2.1)
Completeness (%)	100.0 (100.0)	99.9 (99.9)	100.0 (100.0)
Redundancy	11.9 (12.1)	6.1 (5.7)	5.1 (5.2)
R_merge_	0.200 (0.964)	0.083 (0.622)	0.143(0.734)
**Refinement**			
Number of used Reflections	25,161	29,178	18,983
Protein Nonhydrogen Atoms	5,212	4,943	4,916
Ligand Atoms	81	54	81
Water Molecules	222	171	91
R_work_	0.185	0.173	0.180
R_free_	0.247	0.231	0.259
Average B-factor, (Å^2^)	22.47	25.69	25.82
RMSD from Ideal Geometry			
Bond Length (Å)	0.011	0.015	0.013
Bond Angles (°)	1.41	1.48	1.49

Data collection and refinement statistics for the FAD/SAM-bound ecMnmC, FAD-bound ypMnmC and FAD/SAM-bound ypMnmC. Values for the outer resolution shell of data are given in parentheses.

Subsequent to the release of our structures in the PDB, an independent ecMnmC structure was deposited in the PDB and the associated report of the 3.0Å resolution structure of FAD-bound ecMnmC appeared [14]. In addition to higher resolution (up to 2.30Å), the current work extends these findings by demonstrating the structural conservation of the bifunctional MnmC molecule between species (i.e., *E. coli* and *Y. pestis*), providing the first description of the bi-liganded complexes (i.e., FAD and SAM) from both *E. coli* and *Y. pestis* and by offering a detailed mechanistic analysis of the two reactions catalyzed by this bifunctional enzyme.

### N-terminal domain of MnmC

**Figure 3:**
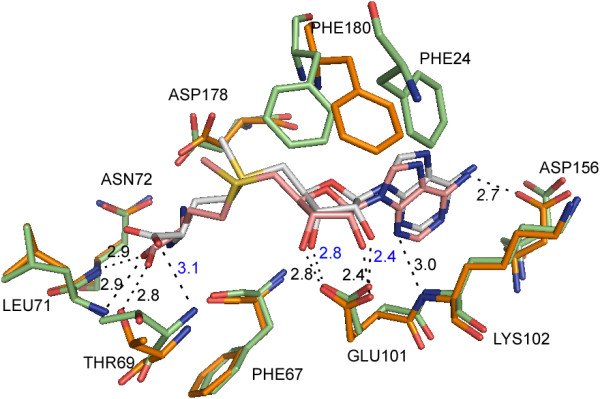
**Comparison of the MnmC2 active sites between ecMnmC and ypMnmC. **Amino acid residues from ecMnmC are displayed in light green, where those from ypMnmC are in orange. Note that all displayed residues are conserved in both proteins except for Phe-24, which is disordered in the ypMnmC structure. EcMnmC bound SAM is colored as light grey (carbon), blue (nitrogen), red (oxygen), and yellow (sulfur), whereas ypMnmC bound SAM is presented in an identical color scheme except for pink (carbon). Hydrogen bonding interactions are shown as black dashes in both structures, where H-bond distances are shown in Å and labeled in blue or black for ecMnmC, or ypMnmC, respectively.

The N-terminal MnmC2 domain is composed of residues 1–245 and contains the SAM binding site. Our structures show that ecMnmC and ypMnmC bind SAM in an almost identical manner (Figure 3). The ~30 disordered N-terminal residues in the ypMnmC structures are near SAM binding site; however, this conformational flexibility does not appear to affect SAM binding, as the ligand was fully occupied in crystals of ypMnmC which had been soaked in mother liquor containing 5 mM SAM (Figures 2 and 3). Importantly, the recombinant ypMnmC is catalytically active, as shown in Additional file 1: Figure S2.

The binding pocket for SAM in MnmC is composed of mostly hydrophobic residues, except for Glu-101 and Asp-178 in both ecMnmC and ypMnmC. Glu-101, which is nearly invariant among SAM-dependent methyltransferases, is engaged in hydrogen bonds, involving its acidic side chain, with the 2′- and 3′- OH groups of the ribose moiety of SAM. Asp-178 is also highly conserved among bifunctional MnmC (>99%), and forms a polar interaction with the amino group of SAM via its acidic side chain. Most interactions between SAM and the protein occur through van der Waals contacts and hydrogen bonds with backbone carbonyl groups, including Phe-67, Thr-69, and Ile-157 in the ecMnmC structure. Although the overall architecture of the binding pocket is highly similar, a few additional residues are found within hydrogen bonding distance from SAM in ypMnmC; *e.g*., Thr-69, Asn-72, Asp-156, and Asp-178, which interact via their side chains, and Leu-71, Asn-72 and Val-157 via backbone amide group (Figure 3).

### C-terminal domain of MnmC

**Figure 4:**
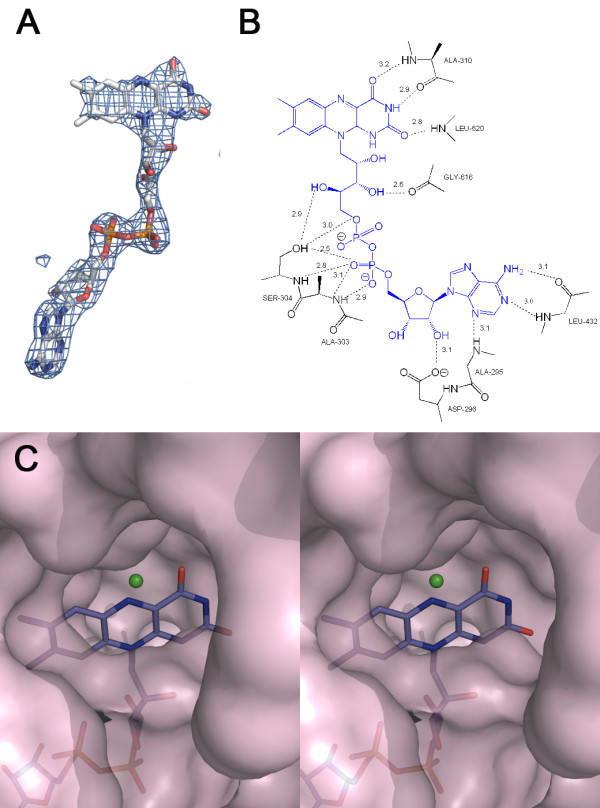
**FAD-binding site in MnmC1 domain. A**) Omit electron density map (Fo-Fc; contoured at σ=3.0) around FAD in ecMnmC. **B**) Schematic diagram showing hydrogen bond interactions of FAD within the active site of ecMnmC within 3.2 Å. Numbers represent distance in Å **C**) Stereo view of the surface representation around ecMnmC1 active site with bound FAD displayed in sticks. Green sphere represents a chloride ion.

All three MnmC structures unambiguously exhibited bound FAD (Figure 4A), even though the cofactor was not added during purification or crystallization. FAD interacts extensively with the C-terminal ecMnmC1 domain through 16 hydrogen bonds with side chain and backbone atoms of 10 residues within 3.2 Å (Figure 4B). Notably, Ser-304 appears to be critical as it is positioned to make multiple hydrogen bonding interactions in both ecMnmC and ypMnmC. This serine residue is conserved in most bifunctional MnmC (136/141), and in all 11 monofunctional MnmC1 enzymes surveyed (Additional file 1: Figure S3). In addition, a chloride ion was modeled near the flavin ring in both ecMnmC and ypMnmC. Electron density for this feature was too strong for water, but the lack of coordination to nearby protein residues or water molecules suggest that it is not a metal ion. Chloride does not appear to be directly involved in tRNA binding, as these species approach opposite sides of flavin ring (Figure 4C).

### Domain interface between MnmC1 and MnmC2

**Figure 5:**
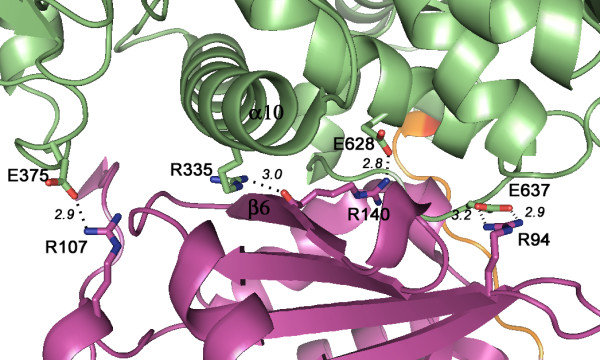
**Domain interface in ecMnmC.** MnmC1 and MnmC2 are purple and green, respectively. The secondary structural elements β6 and α10, located at the core of the interdomain interactions, are labeled. The yellow loop represents the linker region (residues 245–255). Critical atomic interactions at the domain interface, which are described in the text, are shown in Å.

Each domain contributes about 40 residues to the interdomain interface, which buries 1578 Å^2^ of accessible surface area in ecMnmC (Additional file 1: Table S1). In ypMnmC, a smaller solvent accessible surface area of 1241 Å^2^ is buried due to the disordered N-terminal residues. In both ecMnmC and ypMnmC, β6 (residues 137–143 in ecMnmC, and ypMnmC) from the MnmC2 domain and α10 (residues 319–340 in ecMnmC and 319–343 in ypMnmC) from the MnmC1 domain form the core of the interface (Figure 5). The remainder of the interdomain interactions involves residues within loops or turns from each domain, including segments 130–136, 144–147, and 163–168 in the ecMnmC2, and 284–287, 371–375, and 637–643 in the ecMnmC1 domain. Corresponding interdomain interactions in ypMnm involve residues 130–136, 144–147, 163–168 in ypMnmC2, and 284–287, 374–378, and 649–655 in ypMnmC1. A number of polar residues from both domains are capable of participating in hydrogen bonding interactions as shown in Figure 5 and Additional file 1: Table S2. In addition, there are three pairs of ionic interactions in ecMnmC; *e.g.*, Arg-94/Glu-637, Arg-107/Glu-375, and Arg-140/Glu-628; only the Arg-140/Glu-640 interaction is present in ypMnmC.

## Discussion

The crystal structures of ecMnmC and ypMnmC reveal the overall architecture of the full-length bifunctional MnmC, as well as a detailed description of the determinants responsible for binding the SAM and FAD cofactors in the two active sites. YpMnmC and ecMnmC share 61% overall sequence identity and exhibit similar structural organization with an RMSD of 1.1 Å calculated over 626 C_α_s (Figure 2C). All of the current structures exhibit FAD bound in the MnmC1 active site, consistent with the previous identification of this cofactor by TLC analysis [15]. The binding mode of SAM within the MnmC2 domain was examined by soaking pre-existing crystals, which did not elicit any significant structural alteration of the MnmC2 domain or reorganization of the MnmC1/MnmC2 interface. The architecture of MnmC clearly defines the arrangement of the two domains, which separates the catalytic sites for FAD-dependent oxidation and SAM-dependent methylation by approximately 45 Å.

### Evolutionarily conserved residues within MnmC

**Figure 6:**
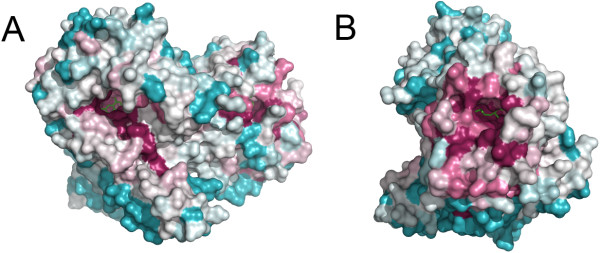
**Evolutionarily conserved amino acid residues among MnmC. **Conservation of each residue is mapped onto the ecMnmC structure where magenta represents highly conserved amino acids and light blue reflects variable regions. **A**) FAD binding site is centered where the cofactor is represented in green sticks. **B**) Enzyme is oriented to display the SAM binding site, where the ligand is shown in green sticks. Conservation score was calculated and presented by the ConSurf server [16].

**Figure 7:**
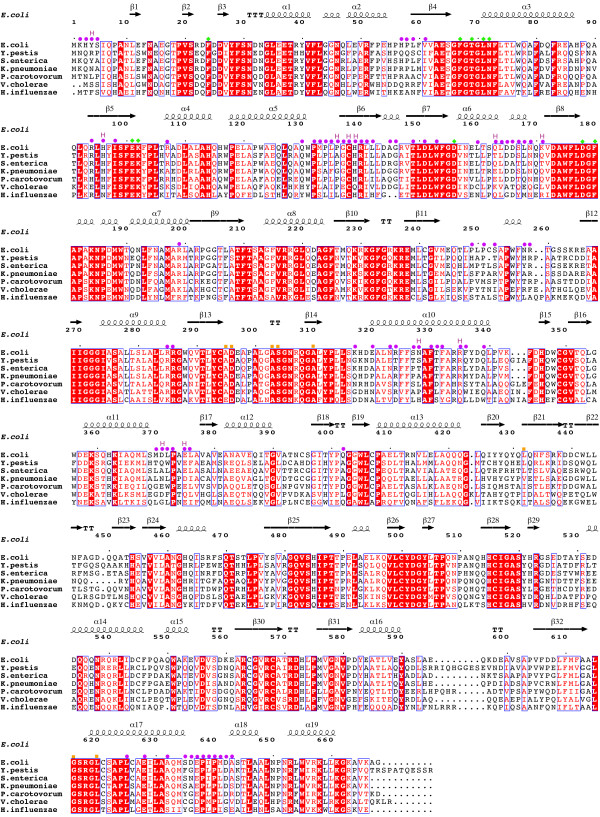
**Multiple sequence alignments of bifunctional MnmC. **Amino acid seuences of seven bacterial MnmC **(**Escherichia coli, Yersinia pestis, Salmonella enterica, Klebsiella pneumoniae, Pectobacterium carotovorum, Vibrio cholerae, and Haemophilus influenza) are aligned and compared. Residue numbering and secondary structural elements are based on ecMnmC. Residues contributing to the interdomain interface in Table 2 are marked in purple circles, where purple H denotes those residues which are involved in hydrogen bonds at the interface. Green diamonds highlighted the residues shown in Figure 3, which form the binding pocket for SAM in ecMnmC. Residues shown in Figure 4 which form hydrogen bonds with FAD are labeled with orange squares.

The National Center for Biotechnology Information (NCBI) data base identifies ~1,200 bifunctional MnmC orthologs (> 80% amino acid sequence coverage of ecMnmC), which are found predominately among the γ-proteobacteria, as well as in some α-, β-, and ϵ-proteobacteria. The mapping of conserved residues onto the ecMnmC structure reveals highly conserved surfaces involved in recognition of the FAD and SAM cofactors, as well other regions in close proximity to the active sites, which are likely to be involved in tRNA binding (Figures 6 and 7).

There are 18 residues that are conserved in greater than 98% of the 140 bifunctional MnmC surveyed (Table 2). Of these, nine cluster around the SAM binding site, with three being near the FAD binding site. The function of the remaining six residues is not entirely clear, although Leu-95 and Gly-287 are located near the interdomain interface, presumably playing a structural role in maintaining the domain-domain interface. Interestingly, the residues at the domain interface are generally not highly conserved, with only two residues, Gly-137 and Leu-625, exhibiting greater than 90% conservation (Additional file 1: Table S1). The interdomain linker (245–255) is one of the most divergent segments.

**Table 2:** Amino acid residues of evolutionary and functional importance

**Residue**	**Function**
Phe-67	SAM binding
Gly-69	SAM binding
Gly-70	SAM binding
Leu-95	Near interdomain interface
Asp-178	SAM binding
Gly-179	SAM binding
Pro-182	SAM binding
Leu-220	unknown
Cys-224	unknown
Lys-236	RNA binding
Gly-271	SAM binding
Gly-273	SAM binding
Gly-287	Near interdomain interface
Gly-518	FAD binding
Arg-567	FAD binding
Gly-578	unknown
Gly-619	FAD binding
Arg-653	unknown

Residues that are conserved greater than 98% among 140 bifunctional MnmC. The numbering is based on the ecMnmC amino acid sequence.

### Surface electrostatic potential of the ecMnmC structure

**Figure 8:**
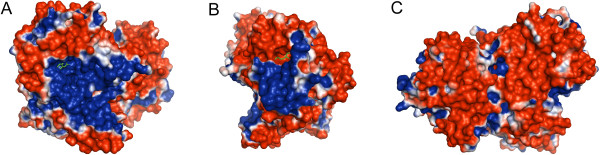
**Electrostatic potential of the protein surface mapped on the ecMnmC structure. **Positive or negative electrostatic isosurface was plotted at contour levels of + kT/e (blue), or – kT/e (red), respectively. **A**) FAD binding pocket, with FAD represented as green sticks, **B**) SAM binding pocket with SAM in green stick. **C**) The protein is oriented with no active site visible. Note that this surface is mostly electronegative in contrast to **A**) and **B**).

To illustrate the charge distribution on the surface of MnmC, electrostatic potential was mapped onto the surface of the ecMnmC structure (Figure 8) [17]. This mapping highlights the fact that the protein surface is predominately negatively charged, with positive electrostatic potential concentrated around the active sites. This distribution supports a mechanism in which the basic patches surrounding both active sites facilitate interactions with the large polyanionic substrates and in particular with the negatively charged phosphodiester backbone proximal to cmnm5s2U34 or cmnm5U34 in tRNA substrates. Notably, these patches of electropositive potential significantly overlap with the highly conserved residues around the ligand binding sites described above (Figure 6) and likely serve as the binding platform for tRNA.

**Figure 9:**
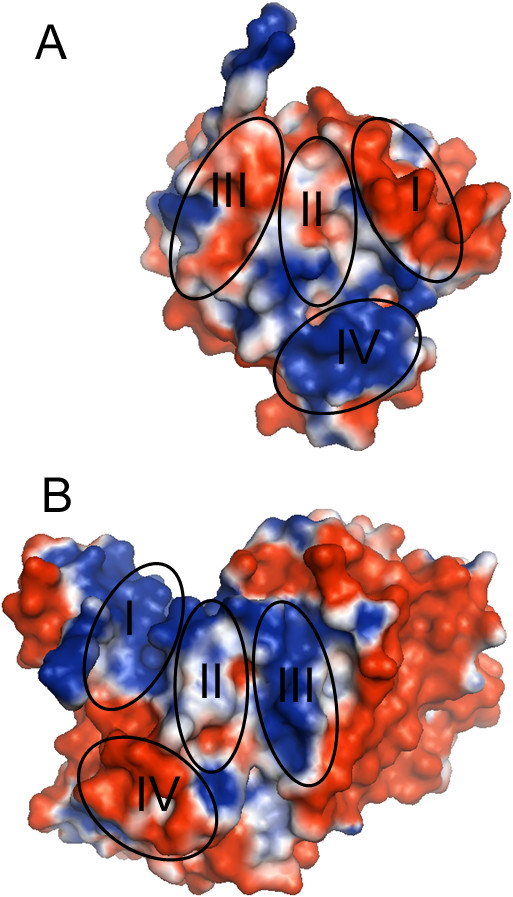
**Charge complementarity between MnmC1 and MnmC2 domains. **Electrostatic potential on the surface contributed to the interdomain interface by **A**) ecMnmC2, **B**) ecMnmC1. The color scheme is identical to that used in Figure 8 Four different patches on each surface were circled and labeled, where a regions with an identical label interact at the interface.

The electrostatics around the interdomain interface is of particular interest, as it highlights the charge complementarity between the two domains. As shown in Figure 9, the interdomain interface may be dissected into four patches on the basis of local physico-chemical properties. The negatively charged patch I in ecMnmC2, which includes residues Asp-165 and Asp-166, interacts with the positively charged patch I in ecMnmC1, largely through backbone amide nitrogen atoms. Patch II, located at the core of the interface contributes primarily hydrophobic interactions. Patch III in ecMnmC1 includes Arg-324 and Arg-335, the side chains of which interacts with the negatively charged Patch III in ecMnmC2, composed mostly of carbonyl oxygen atoms. Glu-628 and Glu-637, contributed by Patch IV of ecMnmC1, interact with the side chains of His-57, Arg-94, and Arg-202 in ecMnmC2. Therefore, the interdomain interface of bifunctional MnmC appears to be maintained through a combination of hydrophobic interactions and electrostatic complementarity between the two domains.

This same strategy for stabilization of the interdomain interface is observed in ypMnmC, as the overall charge distribution on the surfaces contributing to the interface between ypMnmC1 and ypMnmC2 are similar to those in ecMnmC (Additional file 1: Figures S4a and S4b). It is intriguing since most residues at the interface are variable in the bifunctional MnmCs. However, the above mentioned polar residues on the ecMnmC interface surface are mostly conserved in the ypMnmC with exceptions of His-57 and Arg-324, which are replaced with Gln-57 and Thr-324, respectively. It will be interesting to examine whether other bifunctional MnmC employ a similar strategy.

### MnmC1 Is structurally homologous to glycine oxidase of *B. subtilis*

Structural superposition of MnmC1 against the entire PDB returned four independent *Bacillus Subtilis* glycine oxidase structures (PDB codes 1NG3, 1NG4, 1RYI, and 3IF9) as the most similar, with RMSDs of 2.1 - 2.3 Å [18]. EcMnmC1 and glycine oxidase share only 22% sequence identity, with the greatest conservation present in β12 and α9 which contribute to the FAD binding pocket. Glycine oxidase is a required for thiamin biosynthesis in *B. subtilis*, but not in *E. coli*[19], and catalyzes the FAD-dependent oxidation of glycine to iminoglyoxylate, which is trapped by thiocarboxylated ThiS in the next step of the thiazol biosynthetic pathway [20]. *In vitro* assays demonstrate that oxidized glycine is non-enzymatically hydrolyzed to yield ammonium ion, hydrogen peroxide, and glyoxylic acid [21,22], which is analogous to the non-enzymatic hydrolytic reaction associated with MnmC1 (Figure 1).

Intriguingly, the reaction catalyzed by glycine oxidase is highly analogous to the proposed MnmC1-catalyzed reaction [12]. The overall architectures of the MnmC1 domain and glycine oxidase are strikingly similar, as are the organization of the catalytic sites (Additional file 1: Figure S5a). Superposition of the structure of glycine oxidase bound to N-acetyl glycine with ecMnmC (Additional file 1: Figure 5b) provides a model in which the C_α_ of glycine is 3.6 Å away from N5 of flavin in Mnmc1, which is very similar to the analogous distance of 3.5 Å in glycine oxidase. On the basis of the N-acetyl glycine-bound crystal structure and deuterium isotope experiments, a direct hydride transfer mechanism is favored for glycine oxidase, over mechanisms involving formation of a covalent intermediate between flavin and glycine, or mechanisms which are radical-based [19]. At present, it is not clear, however, which oxidative mechanism is utilized by MnmC1. No significant activity was observed when ecMnmC was assayed for the oxidative deamination of glycine or sarcosine (N-methyl glycine) (data not shown). It appears that structural and/or chemical determinants of the modified tRNA substrate are required for turnover by MnmC. It is tempting to speculate that MnmC1, which catalyzes the oxidative deamination of hypermodified wobble uridine (cmnmU34), evolved from glycine oxidase and MnmC2 was subsequently recruited to form a single polypeptide with SAM-dependent methyltransferase activity.

### Mechanistic implications of the MnmC2 active site

SAM-bound structures of both ecMnmC and ypMnmC show that Asp-178 is the only charged residue near SAM, except for Glu-101, which anchors the ribosyl moiety through multiple hydrogen bonds with its side chain. The distance between Cϵ of SAM and Oδ of the Asp-178 side chain is 4.1 Å in ecMnmC and 3.6 Å in ypMnmC. The next closest polar side chain belongs to Asp-26 in ecMnmC, which is 6.6 Å away. Therefore, Asp-178 appears to be the most probable candidate for the Lewis base responsible for activating the amino group of nm5U during the methyl transfer reaction. Both aspartic acid residues are highly conserved based on multi sequence alignments of MnmC. Asp-178 is invariant in both bifunctional and stand-alone MnmC2, while Asp-26 is conserved in 135 out of 140 bifunctional MnmCs and in 48 out of 52 stand-alone MnmC2s.

Phe-67, Gly-68, and Gly-70 are invariant among 140 bifunctional MnmC. These amino acids contribute to the loop connecting β4 and α3, which defines the binding surface for the amino acid portion of SAM. In addition, the side chains of Phe-24 and Phe-180 contact the nucleoside segment of SAM, shielding it from bulk solvent in the ecMnmC structure, although Phe-24 is not visible due to disorder in ypMnmC structure. Phe-24 is conserved as either phenylalanine or tyrosine and Phe-180 is 97% conserved among bifunctional MnmC. Importantly, these structures demonstrate that the reactive donor methyl group is solvent exposed and poised to accommodate the nucleophilic amine of nm5U_34_ (see Figure 1).

### Structural comparison with MnmC2 of aquifex aeolicus

The crystal structure of MnmC2 from *Aquifex Aeolicus* (aaMnmC2; 3VYW) represents a stand-alone MnmC2 that is not fused to an MnmC1 domain [23]. Sequence analysis shows that MnmC2 orthologs exist in a range of bacteria and archea, with most stand-alone MnmC2s being present in *Aquificaceae*, *Cyanobacteria* and *Proteobacteria*. Sequence alignments of aaMnmC2 with ecMnmC2 and ypMnmC2 domains reveal sequence identities of 26% and 27%, respectively.

The overall fold of aaMnmC2 is similar to that of the ecMnmC2 and ypMnmC2 domains, except for approximately 45 residues at the N-terminus and 50 residues in the C-terminus of aaMnmC2 (Additional file 1: Figure S6), which are not highly conserved among monofunctional MnmC2, and the N-terminal ~40 residues are absent in most MnmC2 orthologs (Additional file 1: Figure S3). The RMSD of backbone carbon atoms (Cα) of the aaMnmC2 domain with those of ecMnmC2, and ypMnmC2 are 1.66 and 1.69 Å, respectively (for 199 and 176 Cαs, respectively). In addition, the aforementioned critical beta sheet strand (β6) that contributes to the interdomain interface in ecMnmC is absent in the structure of aaMnmC2. The major structural difference relative to ecMnmC and ypMnmC is in the C-terminal domain loop starting at Ala-261, which corresponds to the C-terminal residue of interdomain linker in ecMnmC (Additional file 1: Figure S6b). In aaMnmC2, this loop extends in a direction nearly opposite that of the linker in ecMnmC and completes a loop-helix-loop-helix motif, which is absent in the ecMnmC and ypMnmC structures. The N-terminal segment of aaMnmC2 also exhibits distinct features, as it contributes residues that interact with the C-terminal segment (Additional file 1: Figure S6b).

The active site architecture of aaMnmC2 is highly similar to that of ecMnmC and ypMnmC. Glu-101 and Asp-178, which appear to be critical for SAM binding and the methyl transfer reaction catalyzed by ecMnmC and ypMnmC, are also conserved in aaMnmC2. In addition, the highly conserved loop in bifunctional MnmC (Gly-66 to Gly-70) is observed in an almost identical conformation, although Gly-66 and Phe-67 are somewhat variable among other monofunctional MnmC2 sequences (Additional file 1: Figure S3). Thus, despite the low sequence identity, bifunctional MnmC and stand-alone MnmC2 share remarkably similar active site configurations and catalytic strategies.

### Biological significance of bifunctional MnmC

MnmC and its corresponding associated tRNA modification are absent in most higher organisms including mammals, although cmnm5U34 is observed in several mitochondrial tRNAs of from *Saccharomyces cervisiae*, *Ascaris suum*, and *Tetrahymena thermophila*[1]. While 5-methylaminomethylation at the wobble uridine has been found in plants (*Hordeum vulgare* and *Triticum aestivum)* as mnm5s2U34 in tRNA^glu^[1], sequence analysis has failed to identify an MnmC ortholog in these organisms. However, identification of MnmC1 or MnmC2 may be difficult in some genomes given the significant sequence divergence exhibited by the methyl transferase and oxidoreductase superfamilies [12]. The structures of both ecMnmC and ypMnmC demonstrate that the two active sites are separated by ~45 Å apart, with the MnmC1 and MnmC2 domains appearing to be rigidly fixed by a substantial interdomain interface that likely precludes direct interactions between MnmC1 and MnmC2 active sites. Thus, if the present crystal structures represent the catalytically active conformation of MnmC, tRNA is likely released to the bulk solvent after oxidation at the MnmC1 active site in order to bind the MnmC2 active site for subsequent methylation.

There are a number of examples of bifunctional enzymes that utilize solvent protected molecular channels to shuttle intermediates between distant active sites [24-27]. Such channeling process may minimize of the loss of metabolites, protect chemically labile intermediates, and orchestrate multiple sequential reactions [28]. This mechanism appears implausible for MnmC given the bulkiness of its tRNA substrates. Another possibility is the induction of conformational rearrangements in MnmC, which brings the two active sites into close proximity. In this case, tRNA does not have to dissociate from the enzyme during turnover; however, given the apparent rigidity of the interdomain interface, this mechanism seems unlikely.

The occurrence of two active sites within a single polypeptide may prove beneficial in other contexts. The gene encoding a bifunctional enzyme is regulated by a single promoter, which provides a powerful constraint in situations where the coordinated expression of separate activities is required. In addition, the local concentrations of the two active sites are much higher if they are covalently tethered. However, it may not be necessary for MnmC to synchronize the two reactions it catalyzes. It has been shown that 5-aminomethyluridine (nm5U34) can be derived from the fusion of 5-formyluridine and ammonia by the MnmE/MnmG complex in the absence of MnmC, both *in vivo* and *in vitro*[9]. Therefore, MnmC2 may be required to convert nm5U34 not originating from MnmC1. Consistent with this notion are recent kinetic studies demonstrating that the activities of the MnmC1 and MnmC2 domains are independent of one another [13]. The lack of coupling between the two active sites in bifunctional MnmC likely ensures comparable turn over of nm5U34 generated from either MnmE/MnmG or MnmC1. Thus, the organization of bifunctional MnmC may have evolved to efficiently drive formation of the final desired product (mnm5U34), regardless of the origin of nm5U34.

## Conclusions

Direct visualization of SAM and FAD in their respective domains defines their binding determinants and the overall organization of bifunctional MnmC. Of particular importance, the structures of *E. coli* and *Y. pestis* MnmC reveal evolutionarily conserved structural features around the active sites and interdomain interface that contribute to function.

## Methods

### Cloning

*mnmC* genes were amplified from genomic DNA of *E. coli BL21* and *Y. pestis Kim* by PCR and cloned into LIC-pET30a (Novagen) following the manufacturer’s protocol. Plasmids containing the *mnmC* gene were selected and verified by the DNA sequence analysis (Genewiz).

### Purification of native MnmC

*E. coli* BL21 (DE3) cells (Invitrogen) were transformed with vectors harboring the *mnmC* genes, grown in LB containing 50 μg/mL kanamycin at 37°C and induced with 0.5 mM IPTG when the OD_600_ reached approximately 1. Cells were further incubated overnight at 25°C and harvested by centrifugation. The cell pellets were resuspended with Bugbuster (Novagen) at room temperature for 30 min, the lysates centrifuged at 18,500 RPM for 30 min and the supernatants applied to Ni-agarose (Qiagene) columns pre-equilibrated with buffer A (50 mM HEPES, pH 7.5, 150 mM KCl and 10% glycerol). The recombinant protein was eluted with 150 mM imidazole in buffer A and the N-terminal hexa- histidine tag removed by overnight incubation with either thrombin or enterokinase (Novagen) for ecMnmC and ypMnmC, respectively. The tag-free proteins were further purified by size exclusion chromatography on a HiLoad Superdex 200 column (GE) equilibrated with buffer A. Final purity was over 95% as verified by SDS-PAGE analysis. Typical yields were approximately 5 mg per liter of fermentation. Enzyme concentrations was determined spectrophotometrically (ϵ_280_ = 1.46 cm^-1^mL/mg for ecMnmC or ϵ_280_ = 1.43 cm^-1^mL/mg for yp MnmC).

### Purification of selenomethionine *Y. pestis* MnmC

*E. coli* strain B834 (Novagen) was transformed with the ypMnmC expression vector, grown in selenomethionine-containing media (Molecular Dimensions) at 37°C, induced with 0.5 mM IPTG when the OD_600_ reached approximately 1 and further incubated overnight at 25°C. The selenomethionyl-substituted protein was purified in a fashion identical to that described for the native protein.

### Gel filtration experiments

Approximately 1 mg of purified MnmC was applied to a HiLoad 16/60 Superdex75 size exclusion column (GE) pre-equilibrated in running buffer composed of 10 mM Tris–HCl, pH 8.0 and 150 mM NaCl. Chromatography was performed with a flow rate of 1.5 mL/min at room temperature. Bovine albumin and γ-globulin were used for calibration and were chromatographed in a fashion identical to that used for MnmC. Eluted proteins were examined by SDS-PAGE.

### Biochemical synthesis of pre-tRNA^Arg^

For the MnmC activity assay, pre-tRNA^Arg^ was prepared *in vitro* with T7-RNA polymerase. The reaction mixture contained 40 mM Tris–HCl, pH 8.1, 1 mM spermidine, 0.001% (wt/vol) Triton X-100, 10 mM DTT, 1 μM T7 promotor (5′- TAATACGACT CACTATAGG-3′), 1 μM anti-sense template (5′- TGTCCCCTGCAGGAATCGAACCTGCAATTGCCCTTAGTTGGGGCTCGTTATATCCATTTAACTAAGAGGACC TATAGTGAGTGCTATTA-3′), 10 mM dNTPs, and 10 units of T7-polymerase (Sigma-Aldrich) in 5 mL and was incubated overnight at room temperature. The RNA transcript is typically of high purity as verified by running the product on the 20% polyacrylamide TBE gel with 8M urea (Biorad). Three rounds of 1:1 (v/v) phenol extraction were performed with the reaction mixture and the final aqueous phase was loaded on HiPrep Desalting column (GE) pre-equilibrated with running buffer identical to that used for the gel filtration experiments described above. Fractions containing tRNA were pooled, concentrated and stored at -20°C.

### Cloning and purification of MnmE and MnmG

MnmE and MnmG genes were amplified by PCR from *E. coli* genomic DNA and inserted into LIC-pET30a. A purification strategy identical that used for MnmC was employed for MnmE and MnmG.

### Assay

The *in vitro* assay for MnmC activity was initiated by mixing 10 mM Tris–HCl, pH 8.0, 1.6 mM 5-formyl-THF, 3.3 mM glycine, 1.7 mM NADH, 3.3 mM GTP, 10 μM T7-transcribed full-length tRNA^Arg^, 0.27 mg/mL *E. coli* MnmE and MnmG, 100 μM [methyl-^14^C] SAM (Perkin Elmer) with bacterial MnmC (0.2 mg/mL) and incubating overnight at room temperature (total volume is 15 μL). The reaction was quenched by addition of 10 μL of 0.5% trichloro acetic acid (TCA) to a 5 μL aliquot of the reaction mixture. The quenched solution was spotted on DE81 filter (GE) and washed sequentially with 0.5 mL ethanol and 8mL 0.5% TCA. The filter was air-dried, images of the radioactive tRNA recorded on a phosphorimaging plate (Molecular Dynamics) and analyzed using a Molecular Dynamics Storm 860 PhosphorImager System with ImageQuant software.

### Crystallization and structure determination

ecMnmC was crystallized by sitting drop vapor diffusion at 21°C by mixing 1 μL of the protein at 10 mg/mL with 1 μL of reservoir solution (1.8 M tri-Ammonium Citrate, pH 7.0 and 0.5% ethyl acetate) and equilibrating over 0.1 mL of reservoir solution. Crystals were transferred to reservoir solution supplemented with 20% glycerol and 5 mM S-adenosyl L-methionine (SAM) prior to flash-cooling in liquid nitrogen. Selenomethionyl-substituted ypMnmC was crystallized by sitting drop vapor diffusion at 21°C by mixing 1 μL of the protein at 10 mg/mL with 1 μL of reservoir solution containing 0.1 M HEPES pH 7.0 and 30% v/v Jeffamine ED-2001^®^ Reagent, pH 7.0 (Hampton Research). Crystals were cryoprotected by soaking in a drop of reservoir solution supplemented with 20% glycerol. SAM/FAD-bound crystals of ypMnmC were obtained by soaking in mother liquor supplemented with 5 mM SAM and 20% glycerol prior to flash-cooling in liquid nitrogen. All X-ray data were collected on an ADSC QUANTUM 315 CCD detector at the NSLS beam line X29A and processed with HKL3000 [29]. Single wavelength anomalous diffraction data extending to 2.3 Å resolution were collected at the selenium peak wavelength for the selenomethionyl-substituted ypMnmC crystals. Diffraction from these crystals was consistent with space group P2_1_ (a = 65.15, b = 59.57, c = 99.63 Å, β = 99.57°), with one molecule per asymmetric unit. Experimental phases were calculated and an initial model built with PHENIX [30]. Iterative rounds of manual model building with Coot [31] and refinement with REFMAC5 [32] to a resolution of 2.31 Å converged at *R*_*work*_ = 0.171 and *R*_*free*_ = 0.234 for the FAD-bound ypMnmC structure. Diffraction data from the FAD/SAM-bound ypMnmC crystal was consistent with space group P2_1_ (a = 66.09, b = 59.82, c = 100.80 Å, β = 100.18) and the structure was determined by molecular replacement using the FAD-bound ypMnmC as a search model with program MOLREP [33]. Subsequent iterations of manual modeling and refinement to a resolution of 2.7 Å yielded a final model with *R*_*work*_ and *R*_*free*_ of 0.180, and 0.259, respectively (Table 2).

Diffraction data from an FAD/SAM-bound ecMnmC crystal were collected at a wavelength λ=1.075 nm and were consistent with space group P4_1_2_1_2 (a = b = 100.06, c = 159.19 Å), with one molecule per asymmetric unit. Molecular replacement was performed using the FAD-bound ypMnmC structure as a search model with MOLREP [33]. Subsequent model building and refinement was performed with Coot and REFMAC5 [32]. The final model was refined to 2.55 Å with *R*_*work*_ = 0.179 and *R*_*free*_ = 0.246 (Table 2).

### Dali server

The coordinates of ecMnmC was submitted to Dali server (http://ekhidna.biocenter.helsinki.fi/dali_server/) in an attempt to search for structurally related proteins in Protein Data Bank (PDB) [18].

### PDB accession numbers

The coordinates and structure factors for the crystal structures of SAM/FAD-bound ecMnmC, SAM/FAD-bound ypMnmC, and FAD-bound ypMnmC have been deposited in the Protein Data Bank (PDB) under accession code of 3PS9, 3SGL, and 3PVC, respectively.

## Competing interests

The authors declare no competing financial interests.

## Authors’ contributions

JK performed molecular biology, crystallization, structure determination, and biochemical analysis. JK and SCA designed the study, analyzed the data and wrote the manuscript. Both authors read and approved the final manuscript.

## Supplementary Material

Additional file 1 Structural basis for hypermodification of the wobble uridine in tRNA by bifunctional enzyme MnmC. Click here for file
