# Supplementary material for: Structural basis for hypermodification of the wobble uridine in tRNA by bifunctional enzyme MnmC
Source: BMC Struct Biol. 2013 Apr 24;13:5. doi: 10.1186/1472-6807-13-5 (PMC3648344; doi:10.1186/1472-6807-13-5)
Supplement: Additional file 1 — Structural basis for hypermodification of the wobble uridine in tRNA by bifunctional enzyme MnmC. [file 1472-6807-13-5-S1.docx]

**Supplementary Material**

**Structural Basis for Hypermodification of the Wobble Uridine in tRNA by Bifunctional Enzyme MnmC**

**Jungwook Kim and Steven C. Almo**

**Albert Einstein College of Medicine**

**Figure Legends**

**Figure S1.** Analytical Size Exclusion Chromatography of ypMnmC. The elution volume (52.2mL) corresponds to a molecular weight of monomeric MnmC (75kD).

**Figure S2.** Dot blot assay of bifinctional ecMnmC and ypMnmC. 14C-SAM was used as the methyl doner in the presence of in situ generated cmnm5U34-tRNAarg and ecMnmC or ypMnmC. The radioactive image was developed on a PhosphorImager plate and analyzed.

**Figure S3.** Multiple sequence alignments of bifunctional (*E. coli and Y. pestis*) and monofunctional MnmC2 (*Aquifex aeolicus, Anabaena variabilis, Nautilia profundicola, and Methanocaldococcus fervens*). Sequences beyond 250 are truncated for simplicity. Residues conserved in all five proteins are highlighted in red, where highly similar residues are in orange. Amino acids corresponding to Glu-101 and Asp-178 in ecMnmC are marked with a star.

**Figure S4.** Electrostatic potential on the surface of interdomain interface of A) ypMnmC2, and B) ypMnmC1. The color scheme is identical to that used in Figure 10.

**Figure S5.** A) Superposed structures of glycine oxidase (blue) and the C-terminal domain of ecMnmC (red). B) Active site of ecMnmC1 with modeled N-acetyl glycine based on the structure shown in A). The distance between N5 of FAD and Cα of glycine is displayed.

**Figure S6.** A) Superposition of aaMnmC2 (green) and ecMnmC (purple). Yellow region within ecMnmC represents the domain linker loop. Secondary structural elements contributing to the interdomain interface are labled on ecMnmC; these elements are missing in aaMnmC2. B) A 90°-rotated view highlights the structural differences between monofunctional and bifunctional MnmC2. Features absent in bifunctional MnmC are located at the N- and C-terminal region of MnmC2.

**Table S1.** Residues observed at the interdomain interface of ecMnmC. Those conserved >80% among bifunctional MnmC are in bold and percentage of conservation is in parenthesis.

| N-terminal domain | | C-terminal domain | |
| --- | --- | --- | --- |
| Lys 2 | Arg 140 | Pro 251 | Leu 372 |
| His 3 | Leu 141 | Ser 253 | Pro 373 |
| Tyr 4 | Leu 142 | Asn 258 | Glu 375  Leu 376 |
| Ser 5 | Ala 145 | Arg 259 | Gln 402 |
| His 57 | Gly 146 | Arg 285 | **Leu 625** (94%) |
| Pro 58 | Thr 149 | **Arg 286** (88%) | **Glu 628** (86%) |
| Leu59 | Asp 151 | Lys 317 | Ala 632 |
| Val 61 | Trp 153 | Glu 320 | Ser 635 |
| Arg 94 | Leu 160 | Asn 323 | Asp 636 |
| **His 96** (80%) | Gln 163 | Arg 324 | Glu 637 |
| Ile 98 | Leu 164 | Ser 327 | **Pro 638** (88%) |
| Arg 107 | Asp 165 | Asn 328 | Ile 639 |
| Gln 130 | Asp 166 | Phe 330 | **Pro 640** (89%) |
| Trp 132 | Ser 167 | Thr 331 | Met 641 |
| Met 133 | Leu 168 | Phe 332 | Asp 642 |
| Pro 134 | Asn 169 | Arg 335 | Ala 643 |
| Leu 135 | Lys 171 | Phe 336 | Leu 646 |
| Pro 136 | Arg 199 | Gln 339 |  |
| **Gly 137** (91%) | Pro 249 | Met 370 |  |
| Cys 139 |  | Asp 371 |  |

**Table S2.** Interdomain hydrogen bonds between the N-terminal and C-terminal domain within the MnmC structures.

|  | ecMnmC |  |  |  | ypMnmC |  |
| --- | --- | --- | --- | --- | --- | --- |
| N-terminal | Distance (Å) | C-terminal |  | N-terminal | Distance (Å) | C-terminal |
| TYR   4 [  N  ] | 3.1 | ASP 371 [ OD2] |  | GLN 130 [ OE1 ] | 2.5 | GLN 339 [ NH2] |
| ARG  94 [ NH1] | 3.2 | GLU 637 [ OE1] |  | CYS 138 [ O ] | 2.6 | SER 328 [ OG ] |
| ARG  94 [ NH2] | 2.9 | GLU 637 [ OE2] |  | ARG 140 [ O ] | 3.0 | GLU 640 [ OH2] |
| ARG 107 [ NH2] | 2.9 | GLU 375 [ OE1] |  | ARG 140 [ O ] | 2.8 | ARG 335 [ NH2 ] |
| PRO 136 [ O ] | 3.2 | ASN 328 [ ND2] |  | ARG 140 [ NH2 ] | 3.0 | PRO 650 [ O ] |
| CYS 139[ O ] | 3.0 | ASN 328 [ ND2] |  | LEU 142 [ O ] | 2.8 | GLN 339 [ NH2] |
| ARG 140 [ O ] | 3.0 | ARG 335 [ NH2] |  | THR 163 [ O ] | 2.9 | ALA 665 [ N ] |
| ARG 140 [ NH1 ] | 3.0 | GLU 375 [ OE1 ] |  | ASP 166 [ OD2 ] | 2.6 | ALA 253 [ N ] |
| ARG 140 [ NH2 ] | 2.8 | GLU 628 [ OE1 ] |  |  |  |  |
| GLN 163 [ O ] | 2.9 | ALA 643 [ N ] |  |  |  |  |
| LYS 171 [ NZ ] | 2.7 | ASP 636 [ O ] |  |  |  |  |

**Figure S1.**


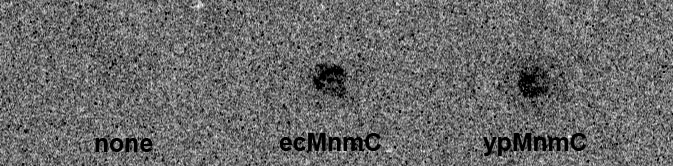


**Figure S2.**


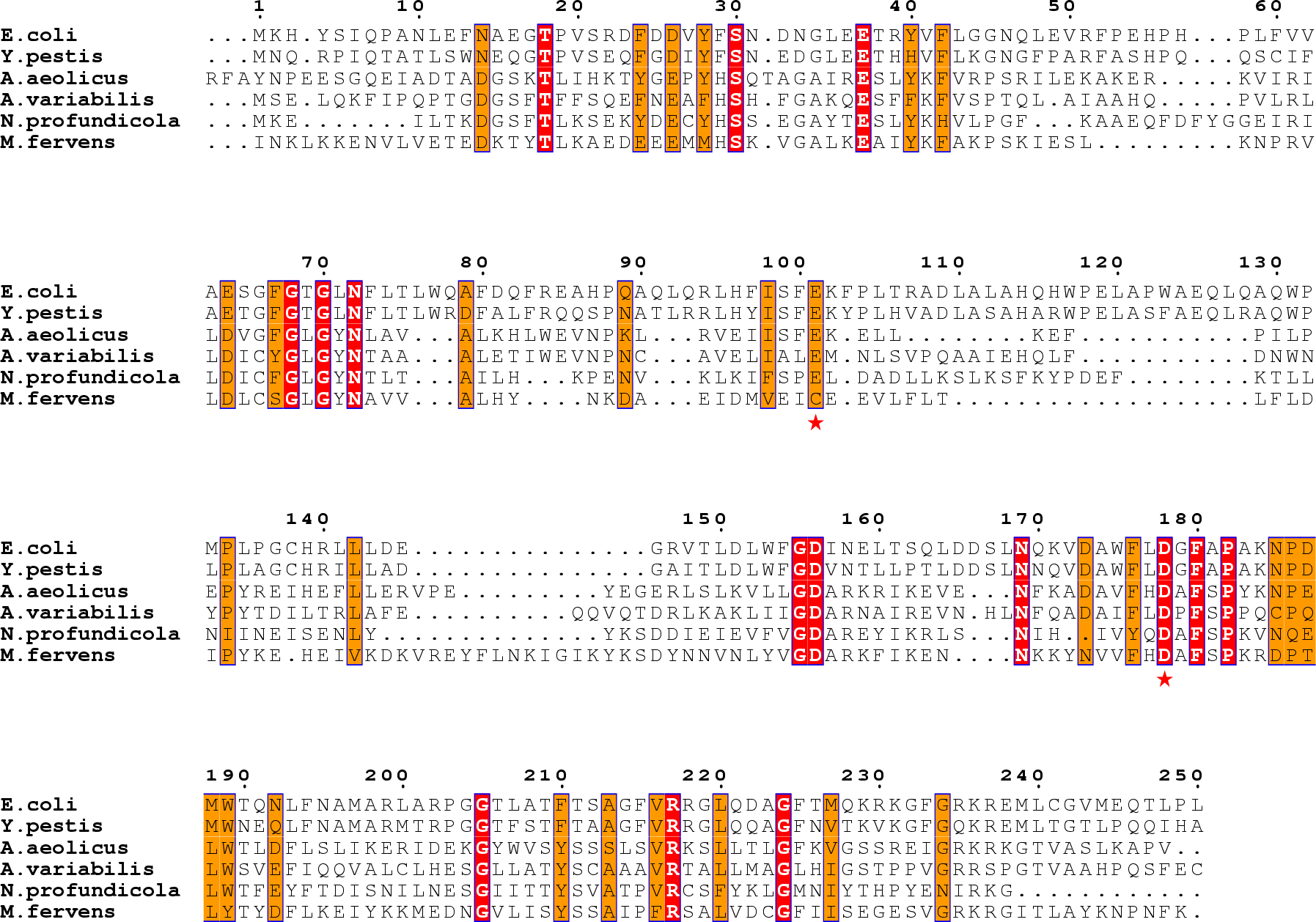


**Figure S3.**

**Figure S4.**


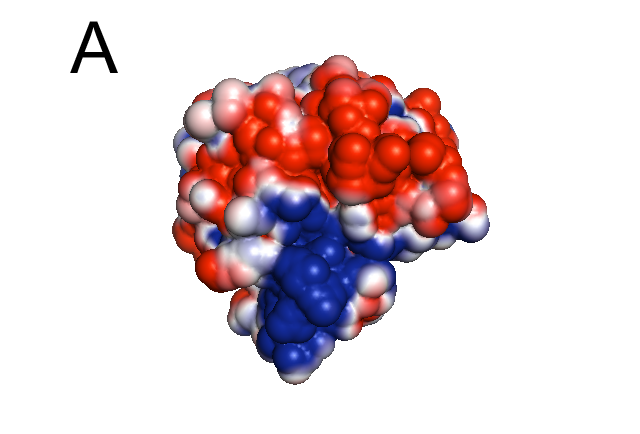

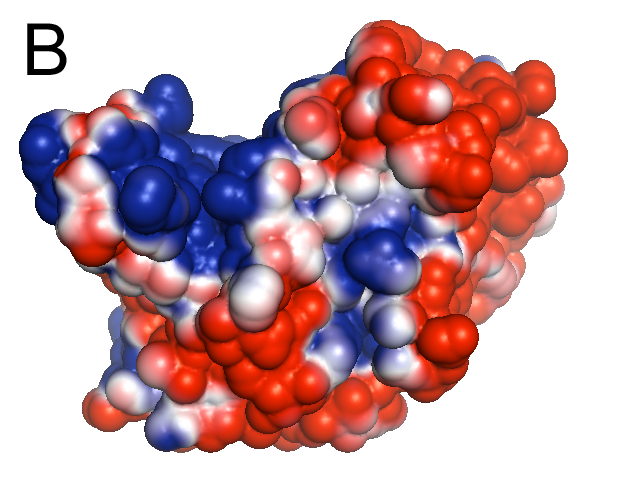


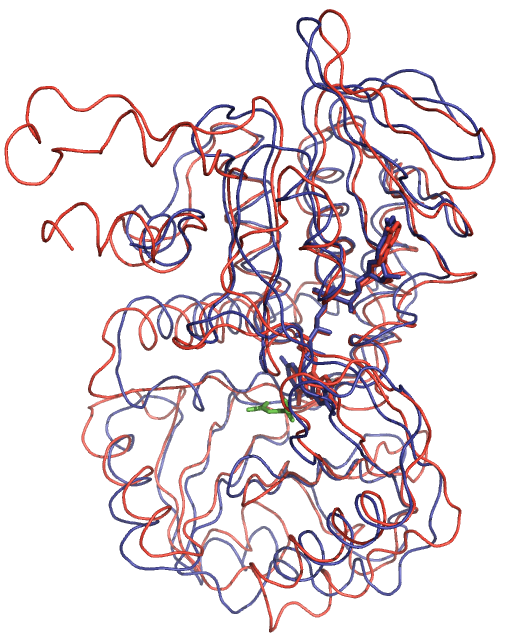


A

B


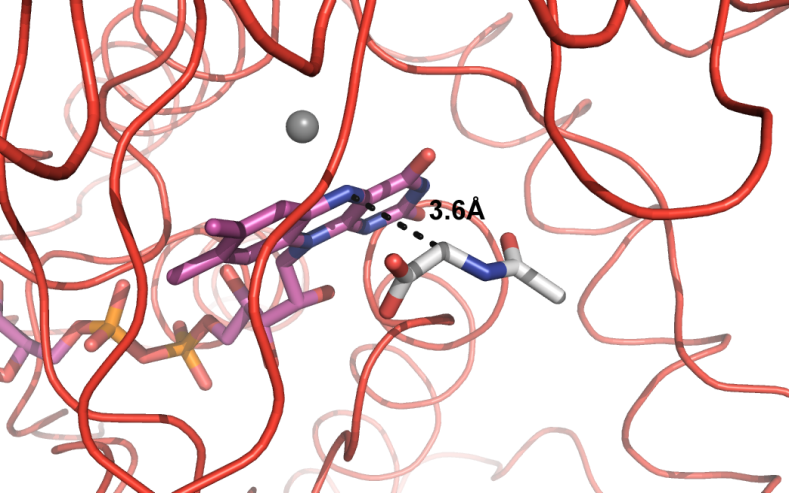


**Figure S5.**


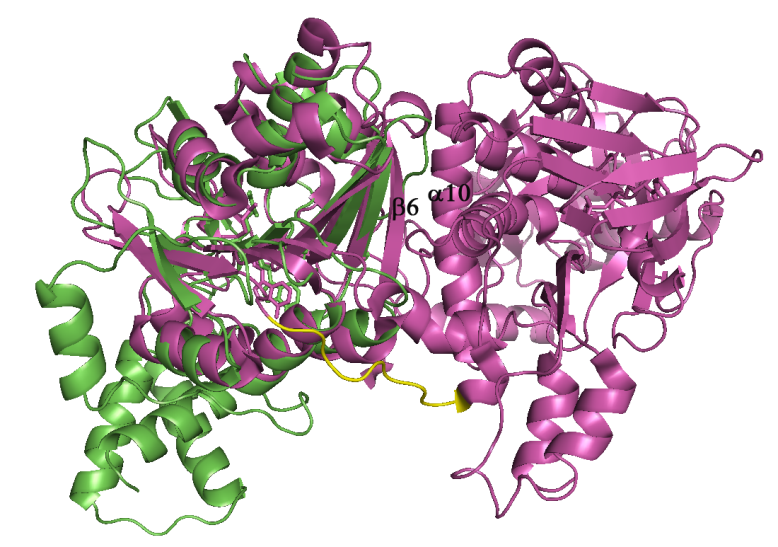


A


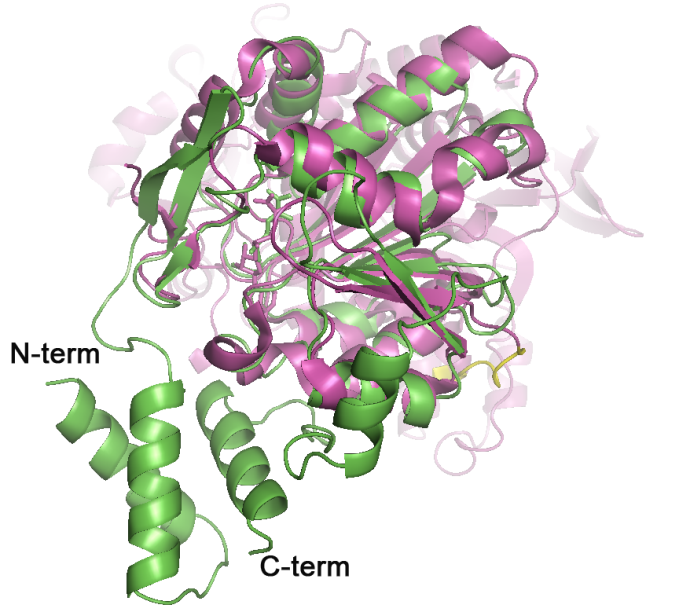


**Figure S6.**

B

**Figure S2.**
